# Co-existent Rhabdoid Tumor of The Kidney and Brain in a Male Infant: A Rare Case

**DOI:** 10.7759/cureus.5423

**Published:** 2019-08-19

**Authors:** Faryal Tahir, Zainab Majid, Laila Tul Qadar, Awais Abbas, Mohammad Raza

**Affiliations:** 1 Internal Medicine, Dow University of Health Sciences, Karachi, PAK; 2 Pediatrics, Dow University of Health Sciences, Karachi, PAK

**Keywords:** rhabdoid tumor, brain, kidney, malignant, infant, atypical teratoid rhabdoid tumors (at/rt)

## Abstract

Malignant rhabdoid tumor of the kidney (MRTK) is a rare neoplasm of infancy. We report a case of a nine-month-old male infant who presented to the pediatrics outpatient department with the history of fever, lethargy, and abnormal head movements. On gross examination, the patient had a firm, non-tender, intra-abdominal mass at the right lumbar region with irregular margins. Computed tomography scan of the abdomen revealed a lobulated soft tissue arising from the kidney with areas of necrosis. Brain magnetic resonance imaging was also performed, which showed a large heterogeneous lesion in the posterior fossa. Histopathologic study revealed loss of INI1 protein. Since MRTK and atypical teratoid rhabdoid tumor (ATRT) of the brain share a common mutation in the gene (hSNF5/INI1), hence a diagnosis of MRTK with co-existent ATRT of the brain was established. Actinomycin-D and vincristine failed to show any improvement and the condition of the patient deteriorated progressively, resulting in his death within 15 days of hospital admission.

## Introduction

Rhabdoid tumor of the kidney (RTK), a rare and highly malignant neoplasm of infancy [1], constitutes only 1.7% of all renal tumors [2]. In 1978, Beckwith and Palmer described malignant rhabdoid tumor of the kidney (MRTK) as a "rhabdomyosarcomatoid variant of Wilm’s tumor" because of the resemblance of cells to rhabdomyoblasts [3]. Subsequently, it was recognized as a unique malignant renal tumor characterized genetically by deletion/mutation of SMARCBI/INI gene on the long arm of chromosome 22 [4]. The exact cell of origin for this tumor is still unknown. However, origin from primitive cells of renal medulla has been considered [5]. Histologically, RTK is characterized by cells arranged either as diffuse sheets or as alveolar or trabecular pattern. MRTK is significantly associated with primary brain tumors or brain metastasis. It usually starts in the kidneys but can also manifest primarily in other soft tissues, especially the brain where it is referred to as atypical teratoid rhabdoid tumor (ATRT). We report a case of a nine-month-old male infant with synchronous RTK and ATRT.

## Case presentation

A nine-month-old male infant presented with the complaints of fever and lethargy for the past one month and abnormal head movements since the last fifteen days. A right-sided abdominal mass was also observed by the mother five days prior to the admission. The head movements were oscillatory, not associated with fits or unconsciousness. Gross examination revealed a firm, non-tender, intra-abdominal mass (measuring approximately 6 x 7 cm) at the right lumbar region with irregular margins and smooth non-erythematous overlying skin. The mass was immobile and abutted on the underlying viscera. The decreased tone in all four limbs and loss of neck holding was observed on central nervous system (CNS) examination. There was no positive history of hematuria.

**Figure 1:**
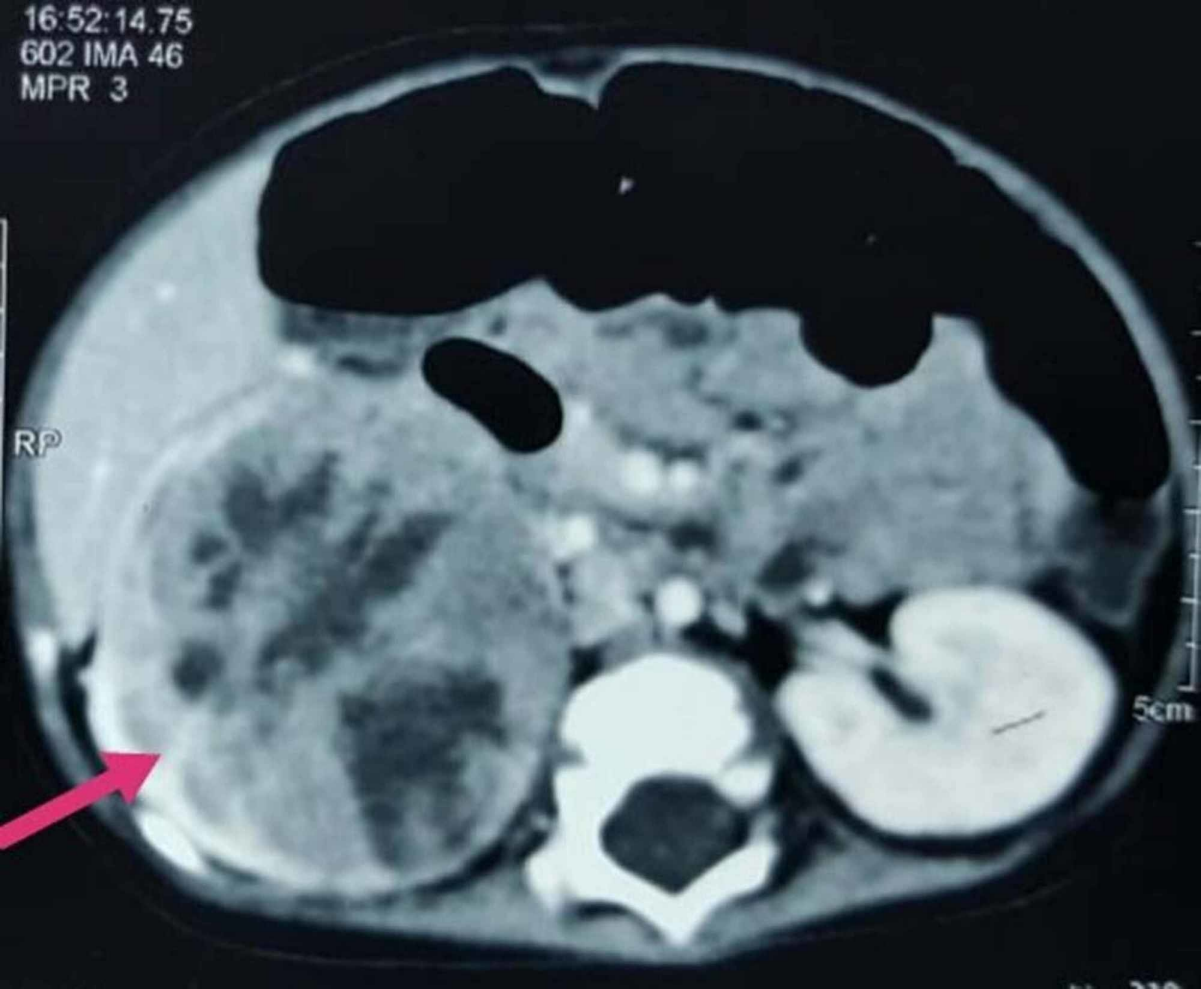
Computed tomography scan of the abdomen showing a huge lobulated mass arising from the right kidney with mild enhancement exhibiting multiple foci of necrosis

Initial laboratory investigations revealed leukocytosis. Abdominal ultrasound (US) demonstrated a solid 6 x 5.3 cm mass at the upper pole of the right kidney with thickened renal parenchyma. On performing computed tomography (CT) scan of the abdomen, a lobulated soft tissue arising from the kidney was seen with areas of necrosis (Figure 1).

**Figure 2:**
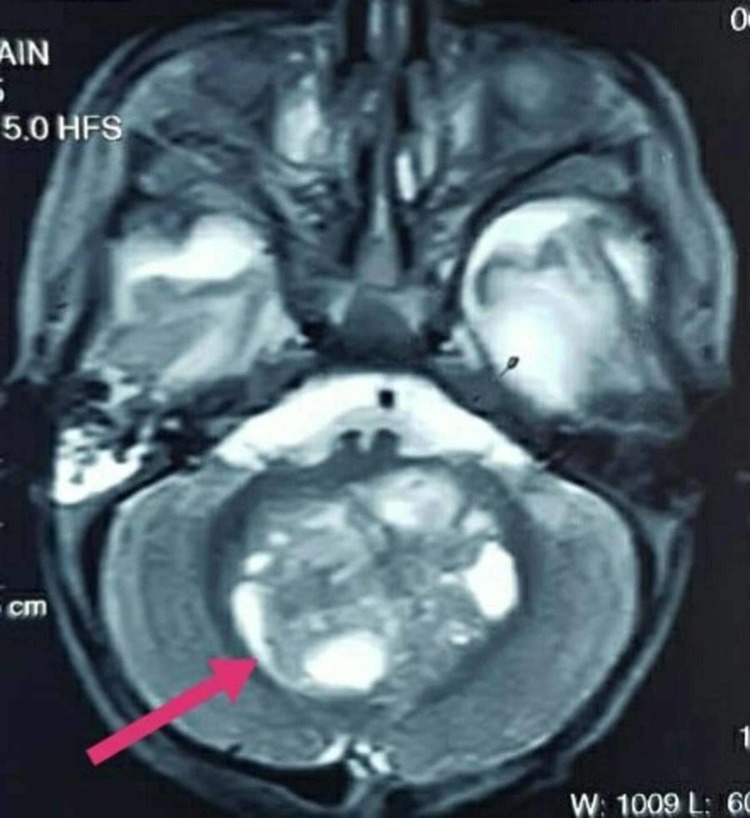
Magnetic resonance imaging of the brain showing a round heterogeneous mass in the posterior fossa along midline

**Figure 3:**
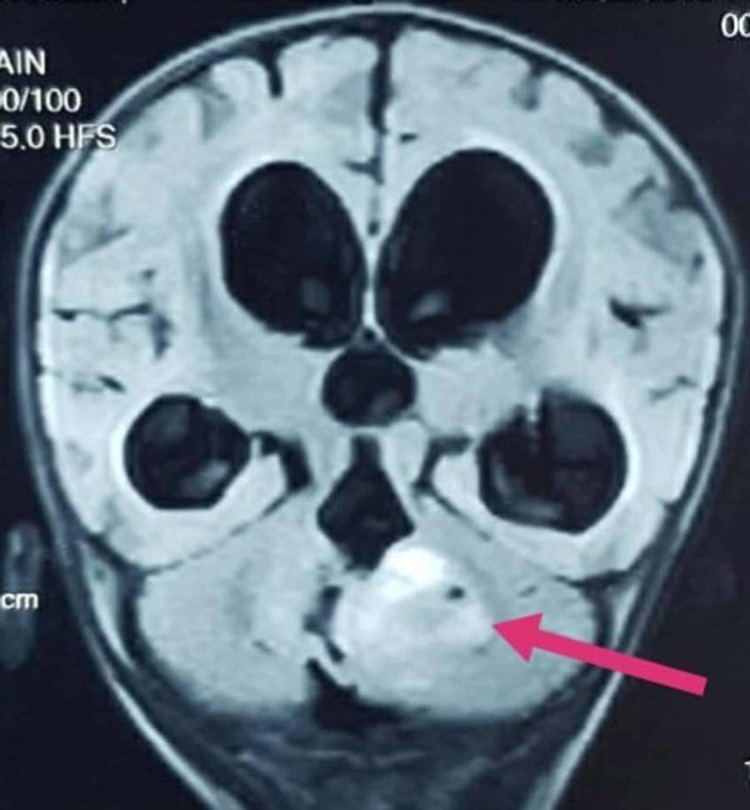
A mass in the posterior fossa compressing fourth ventricle with consequent obstructive hydrocephalus

There was an extension of the mass to the right paracolic region, encasing the right renal vessels along with the invasion of the lymph nodes in the left para-aortic, interaortocaval, and mesentry. The findings were suggestive of Wilms tumor with neuroblastoma being considered as a differential diagnosis. However, the microscopic analysis of the neoplasm displayed sheets of large monomorphic cells with eosinophilic cytoplasm and eccentric, prominent nucleoli. The immunohistochemical study revealed positive immunomarkers for epithelial membrane antigen (EMA), Wilm's tumor transcription factor-1 (WT-1), periodic acid-schiff (PAS), and vimentin. Moreover, histopathological study revealed loss of SMARCB1/INI1 protein expression. Thus, the right MRTK was confirmed. The results of echocardiogram and CT of the chest were unremarkable. Brain magnetic resonance imaging (MRI) displayed a large heterogeneous lesion, measuring approximately 4.7 x 4.2 x 3.7 cm in the posterior fossa (Figure 2) along midline causing compression of the fourth ventricle (Figure 3) and dilation of the third ventricle.

**Figure 4:**
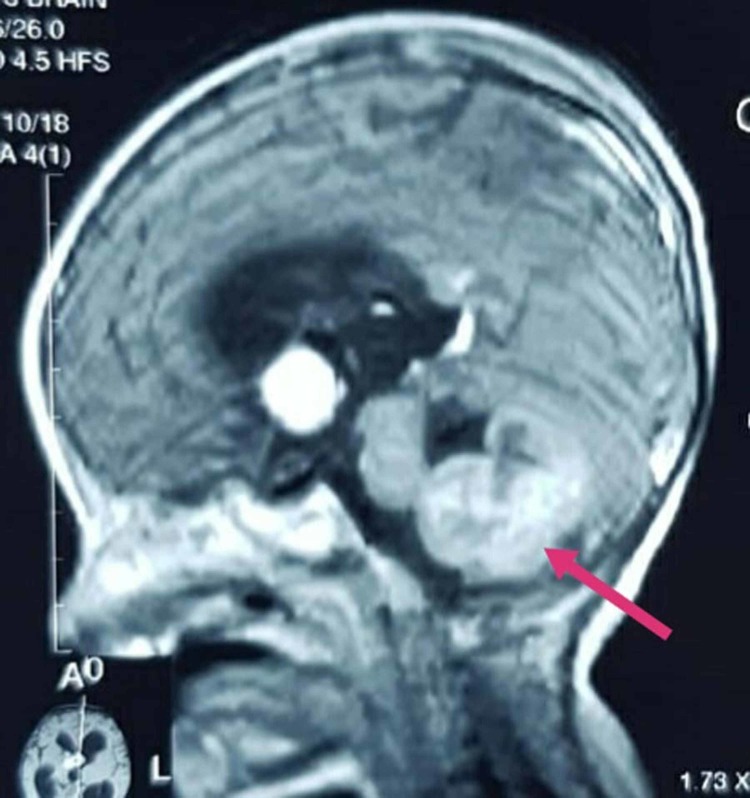
A mass in the region of foramen of monro

Another smaller, solid mass was found in the region of foramen of munro with dimensions of 2.9 x 2.1 cm (Figure 4). As INI1 staining displayed the absence of nuclear staining, hence MRTK with ATRT of the brain was diagnosed since they both share the same mutation of the gene (hSNF5/INI1).

A chemotherapy plan was initiated for the patient, comprising of actinomycin-D and vincristine along with supportive measures. However, no response to the therapy was observed and the patient’s clinical condition deteriorated rapidly resulting in his death within 15 days of presentation. 

## Discussion

RTK, being an aggressive tumor, metastasizes very early to distant regions [6]. Previous studies have shown 22%-28% of the patients with metastatic disease at initial diagnosis and 10%-21% of the cases with CNS involvement [4]. Contrary to this, our patient had primary brain tumor i.e., ATRT along with MRTK. When CNS is affected, raised intracranial pressure produces symptoms of vomiting and decreased levels of consciousness [7] but our patient presented only with abnormal movements of the head. Apart from brain metastasis, CNS involvement in RTK patients can also present with coexistent intracranial neoplasm such as pineoblastoma, primitive neuroectodermal tumor, medulloblastoma, ependymoma, astrocytomy, and germ cell tumor [1]. Thus, recommended intracranial imaging is crucial at the time of diagnosis. In the International Society of Pediatric Oncology, CNS involvement was only seen in 2.8% of the RTK patients because the intracranial evaluation was not a mandatory diagnostic procedure [8]. In accordance with Reinhard et al., only one patient was reported to have synchronous brain lesion among 32 patients of RTK [9] which adds rarity to our case.

MRTK usually affects the pediatric population with an average age of 11 months as evident by our case. Although, diagnosis of any renal mass requires confirmation with histopathologic features. However, a distinct clinical presentation with fever and hematuria in a young age with a high stage tumor at presentation suggests the diagnosis of RTK [6]. Our patient presented atypically with the history of fever but had no complaints regarding hematuria. Presenting only with an abdominal mass is common among adults.

The main differential diagnosis of RTK is the far more common Wilm’s tumor. Even though imaging results appear to be similar, a number of other features can suggest RTK. These features include subcapsular fluid collections, hypoattenuating areas of necrosis and hemorrhage separating tumor lobules, linear calcifications, vascular and local invasion and a concomitant intracranial neoplasm [10] as observed in our case.

Due to the non-specific clinical presentation, it is challenging but also essential to make a definitive diagnosis. The MRTK in the present study was diagnosed mainly on the findings of CT scan aided by immunohistochemical features. The tumor cells of RTK are usually stained positive for vimentin and EMA [11], which is consistent with our case. Furthermore, loss of INI1 protein is highly associated with MRTK and synchronous ATRT of the brain.

Inhibiting fibroblastic growth factor receptors (FGFRs) has been recommended as a novel therapy for malignant rhabdoid tumors (MRTs) [12]. Targeting cell cycle, epigenetic genes, or specifically the molecular profiles of tumor subset may also help in the treatment of rhabdoid tumors (RTs) [13]. Specifically, the treatment of RTK consists of radical nephrectomy along with the resection of surrounding lymph nodes followed by chemotherapy.

RTs have the worst prognosis among all the renal tumors. It depends on the age of the patient at the time of diagnosis and also the stage of the disease. A four-year survival of 8.8% was reported for infants less than six months at the age of diagnosis as compared to 41.1% for children of 2 years [4].

## Conclusions

RTs in the pediatric population have a dismal prognosis, independent from localization. The presence of metastasis at the time of diagnosis appears to be the only prognostic factor. Moreover, due to its significant association with brain tumors or early brain metastases, concurrent brain imaging is essential for all patients.
